# Dietary saturated and monounsaturated fats protect against acute acetaminophen hepatotoxicity by altering fatty acid composition of liver microsomal membrane in rats

**DOI:** 10.1186/1476-511X-10-184

**Published:** 2011-10-20

**Authors:** Jinah Hwang, Yun-Hee Chang, Jung Hwa Park, Soo Yeon Kim, Haeyon Chung, Eugene Shim, Hye Jin Hwang

**Affiliations:** 1 Department of Foods and Nutrition, College of Natural Sciences, Myongji University, YongIn 449-728, Republic of Korea; 2 Research Institute of Food & Nutritional Sciences, Yonsei University, Seoul, 120-749, Republic of Korea; 3 Department of Food and Nutrition, Division of Natural Sciences, Seoul, 100-751, Republic of Korea; 4 National Hypertension Center, Yonsei University Health System, Seoul 120-752, Republic of Korea; 5 Department of Food and Nutrition, College of Human Ecology, Dongeui University, Busan 614-714, Republic of Korea

**Keywords:** Phospholipid fatty acid composition, SFA, MUFA, Hepatotoxicity, Cytochrome P-450 2E1

## Abstract

**Background:**

Dietary polyunsaturated fats increase liver injury in response to ethanol feeding. We evaluated the effect of dietary corn oil (CO), olive oil (OO), and beef tallow (BT) on fatty acid composition of liver microsomal membrane and acute acetaminophen hepatotoxicity.

**Methods:**

Male Sprague-Dawley rats were fed 15% (wt/wt) CO, OO or BT for 6 weeks. After treatment with acetaminophen (600 mg/kg), samples of plasma and liver were taken for analyses of the fatty acid composition and toxicity.

**Results:**

Treatment with acetaminophen significantly elevated levels of plasma GOT and GPT as well as hepatic TBARS but reduced hepatic GSH levels in CO compared to OO and BT groups. Acetaminophen significantly induced protein expression of cytochrome P450 2E1 in the CO group. In comparison with the CO diet, lower levels of linoleic acid, higher levels of oleic acids and therefore much lower ratios of linoleic to oleic acid were detected in rats fed OO and BT diets.

**Conclusions:**

Dietary OO and BT produces similar liver microsomal fatty acid composition and may account for less severe liver injury after acetaminophen treatment compared to animals fed diets with CO rich in linoleic acid. These findings imply that types of dietary fat may be important in the nutritional management of drug-induced hepatotoxicity.

## Introduction

Dietary saturated fatty acids (SFA) in beef and pork are protective against alcohol-induced liver disease in man and animals, whereas polyunsaturated fatty acids (PUFA) from corn oil and fish oil augment hepatic fibrosis and necrosis in rats fed a liquid diet containing alcohol [1-4]. In particular, would a lipid profile similar to the "Mediterranean diet" (low fat, high oleic acid content, lower PUFA and SFA) be protective against liver injury when compared to a high-linoleic acid diet such as the diet consumed by most U.S. citizens or the AIN93 rodent diet with 7% soybean oil? This is plausible because monounsaturated fatty acids (MUFA) are more readily incorporated into membrane phospholipids than are SFA. The major change in tissue phospholipids when these diets are fed is a prominent decrease in linoleic acid and an increase in oleic acid [5].

The cytochrome P450 (CYP)-dependent liver microsomal mixed function oxidase (MFO) system, which exists in membrane vesicles of the endoplasmic reticulum, plays an important role in the metabolism of various drugs and foreign compounds. This system is responsible for activating acetaminophen in the liver to an electrophilic intermediate that can bind covalently to cellular macromolecules to produce cell damage [6]. The activity of this system affected by nutrients and lipid is one of the important factors because dietary fats can be reflected in the fatty acid composition of membrane phospholipids and thus alter the membrane conformation and fluidity, resulting in changes in the MFO activity [7]. Increased PUFA in microsomal membranes enhance xenobiotic metabolism. The changes in the fatty acid composition of the membrane phospholipids alter the conformation of CYP, and this could alter the presentation of its active binding site and therefore the metabolism of xenobiotic substrates [8].

Although diets that contain SFA and possibly MUFA may protect the liver against toxic agents, it is unlikely that any public agency would recommend an increase in intake of animal fats. In this study, a rat model of hepatotoxicity was used to test the hypothesis that diets with MUFA (olive oil) prevent liver injury as well as SFA (beef tallow) after acetaminophen challenge when compared to diets with PUFA (corn oil). To fully determine the effect of dietary fats on liver injury and cell death, male rats of three groups were fed isocaloric diets with different fat sources for 6 weeks. One set of animals in each group was challenged with acetaminophen.

## Methods

### Animals and diets

Thirty-six male Sprague-Dawley rats (Charles River, NC, USA) weighing 150 g to 190 g were housed individually in hanging wire-mesh cages with feeding cups. The room temperature (21 ± 1°C), lighting (on at 06:00, off at 18:00), and humidity (40-50%) were controlled. Rats had free access to restricted experimental diets and tap water.

Animals were randomly assigned to three diet groups and fed the modified AIN 93G diet for 6 weeks. Rats in group 1, 2 and 3 were fed 15% (wt/wt) corn oil, olive oil and beef tallow diets, respectively. All diets also contained: Casein, 200 g/kg; sucrose, 100 g/kg; celufil (Solka-Floc), 50 g/kg; mineral mix (AIN-93G-MX), 35 g/kg; vitamin mix (AIN-93-VX), 10 g/kg; choline bitartrate, 2.5 g/kg; L-cystein, 3 g/kg; and tert-butylhydroquinone, 0.014 g/kg. The semipurified control diet was fed for one week to acclimate the animals to feed cups and individual caging. Fatty acid composition of each diet is shown in Table 1.

**Table 1:** Fatty acid composition of corn oil, olive oil and beef tallow

Fatty Acid	Corn oil	Olive oil	Beef tallow
	
		μmole %	
C14:0	-	-	2.3
C16:0	8.3	10.8	29.8
C18:0	2.5	2.9	23.3
C18:1n-9	23.5	67.8	34.2
C18:2n-6	59.8	9.7	2.7
C18:3n-3	1.32	0.4	-

ΣSFA	10.8	13.7	55.4
ΣMUFA	23.5	67.8	34.2
ΣPUFA	61.1	10.1	2.7

Each dietary group was subdivided into case and control groups. Each case group (n = 6) was unfed overnight prior to acetaminophen challenge. Acetaminophen was given intraperitoneally in a vehicle of gum arabic (2% in saline) at a dose of 600 mg/kg body weight. For 7 hr afterwards, rats were anesthetized with ketamine: acepromezine: xylazine (3:2:1) at a dose of 0.1 mL/100 g body weight and blood samples were collected by cardiac puncture in tubes containing EDTA and centrifuged at 2, 500 rpm for 20 min for liver function tests. The animals then were perfused transcardially with 0.9% NaCl and livers were snap-frozen. All frozen samples were stored at -80°C for later experiments. All experimental procedures were approved by the Ethics Committee and the Institutional Animal Care and Use Committee of the facility.

### Measurement of plasma GPT and GOT

Glutamate-pyruvate transaminase (GPT) and glutamate-oxaloacetate transaminase (GOT) were measured by the Sigma transaminase [ALT/GPT and AST/GOT] Diagnostic kit (Sigma Chemical Company, St. Louis, MO, USA). A standard curve was prepared using the Calibration standard solution with six levels of pyruvic acid. In brief, 0.2 mL plasma was added to 1.0 mL Sigma Prepared Substrate for GOT or 1.0 mL Alanine-alpha-KG Substrate for GPT in a 37°C water bath for 1 hr and 30 min, respectively. Then 1.0 mL Sigma Color Reagent was added at room temperature for 20 min, followed by adding 10.0 mL 0.40 N NaOH solution with vigorous mixing. Absorbance at 505 nm was recorded and the enzyme activities were determined in Sigma-Frankel (SF) Units/mL from the calibration curves.

### Measurement of hepatic GSH

For measurement of reduced glutathione (GSH), a standard curve was prepared using reduced GSH in 0.02 M EDTA. The curve was linear between 3.0 and 100.0 nmols GSH and data were expressed as μ mole GSH per g tissue. Briefly, 600 μL aliquots of liver homogenate were treated with 60 μL 55% trichloroacetic acid, shaken well and allowed to stand on ice for 10 min, followed by centrifugation at 4, 000 g for 12 min. Four hundred μL of the supernatant was mixed with 1.0 mL of 1.2 M Tris in 0.06 M EDTA (pH 8.9) and 100 μL of Ellman's reagent (DTNB). The samples were allowed to develop at room temperature for 15 min and absorbance at 412 nm was recorded.

### Lipid peroxidation

Lipid peroxidation was evaluated by thiobarbituric reactive substances (TBARS). In brief, frozen hepatic tissue samples were homogenized in ice-cold phosphate buffer (0.05 M, pH 7.4) and immediately treated with thiobarbituric acid and placed in a boiling water bath for 12 min. This procedure was followed by butanol extraction and subsequent reading of the supernatants at 532 nm. The equivalent malondialdehyde (MDA) concentration was calculated on the basis of the molar extinction coefficient of the malondialdehyde-thiobarbituric acid complex (1.56 × 10^5 ^M^-1 ^cm^-1^). A standard curve was prepared using 1, 1, 3, 3-tetraethoxypropane. The curve was linear between 0.01 and 1.5 nmoles of MDA. Data are expressed as nmole MDA per mg protein.

### Preparation of liver microsomes

The right lobe of liver was suspended in 25% homogenizing buffer in ice-cold 1.15% KCl/50 mM Tris-HCl/1 mM EDTA (pH 7.4), homogenized and centrifuged at 10, 000 g at 4°C for 20 min. The resulting supernatant was centrifuged at 105, 000 g for 1 hr at 4°C and the microsomal pellet was suspended in 0.1 M potassium phosphate buffer (pH 7.4).

### Western blot analysis

Liver microsomes were resolved in 10% SDS-polyacrylamide gel electrophoresis, transferred onto a nitrocellulose membrane (Whatman), and probed with primary antibodies specific to CYP 2E1 (Amersham) and actin (Santa cruz). After washing, membranes were incubated with horseradish peroxidase-conjugated secondary antibody. Immunodetection was performed using a chemiluminescence method (SuperSignal, Pierce) and relative densities of bands were quantified using an NIH image J program http://rsb.info.nih.gov/ij/. All data were normalized to the actin content of the same sample.

### Fatty acid analysis

Extraction of fatty acids in phospholipids of liver microsomal membranes and plasma was performed using the modified method of Folch *et al*. [9]. Extracted fatty acids were applied to the silica gel 60 TLC plate (Sigma). After the application, solvent was allowed to dry and the plate was fully developed to 18 cm in acetone/acetic acid/water (100:2:1, by vol.) for phospholipid. The lipid classes were visualized by spraying the plates with rhodamine 6G (0.02% in 95% ethanol) and exposing the plates to ultraviolet light. All of the silica in representative bands was scraped into 10 mL, screw-capped tubes (Teflon-lined caps). All of the silica in the phospholipid band was added to 3 mL 6% HCL in methanol and 40 μL internal standard (heptadecanoic acid, C 17:0, Nu Chek, Elysian, MN, USA) and transmethylated at 75°C for 2 hr. The tubes were cooled on ice, 2 mL of hexane and 1 mL KCl were added and the tubes were vortexed for 1 min and centrifuged at 1, 000 rpm for 10 min. The hexane extract (upper phase) was passed through a sodium sulfate column. Samples were concentrated under a stream of nitrogen for gas chromatograph (GC) analysis.

Fatty acid analysis was performed with a 5890 GC (Hewlett-Packard) using a flexible fused capillary column (30 m × 0.25 mm I.D., thickness 0.25 μm, J & W Scientific, Folsom, CA). The column temperature was programmed to begin at 205°C for 20 min and then increase at a rate of 3°C/min to a final temperature of 220°C, which held for 3 min. The injection port temperature was 250°C and the detector was 260°C. Fatty acid values were presented as μmole % which represents the area under the chromatograph identified as a particular fatty acid based on its retention time when compared to the internal standard.

The double bond index (DBI) was calculated with the results of fatty acid analysis. DBI equals the sum obtained by multiplying the fraction of each fatty acid times the number of double bonds contained and adding the total together [10].

### Statistical analysis

All data were presented as mean ± SD. The data were analyzed with nonparametric methods due to small sample sizes using SAS computer-based statistics programs. Kruskal-Wallis test and the Wilcoxon rank sum test were performed for one-way analysis of variance and statistical differences between two groups, respectively.

## Results

### Body and liver weight

There were no significant differences in the initial and final body weights which increased approximately from 150 g to 330 g after 6 weeks feeding period. Liver weights were not significantly different among groups (data not shown).

### GPT and GOT concentrations

**Figure 1:**
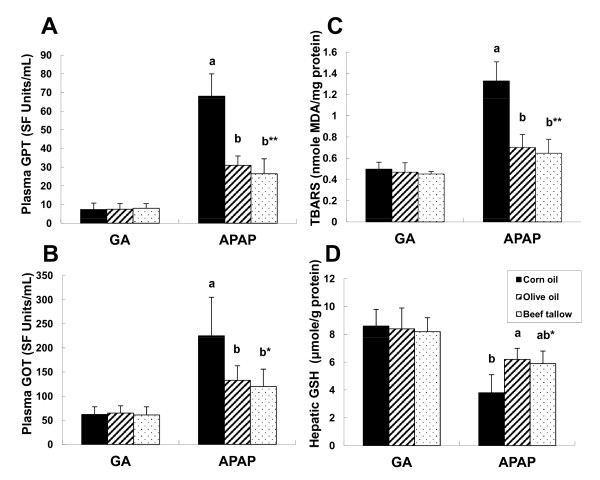
**The effect of acetaminophen on GPT (A), GOT (B), TBARS (C) and hepatic GSH (D) levels**. Abbreviations: GA, gum arabic vehicle injected to control group; APAP, acetaminophen injected to case group. Each bar represents the mean ± SE (n = 6). Different letters (a & b) indicate significant difference among APAP groups (* p < 0.05, ** p < 0.01)

Acetaminophen-induced changes in plasma GOT and GPT were significantly enhanced in corn oil fed rats, compared to beef tallow and olive oil fed animals (Figure 1A &1B). GPT and GOT levels were increased by approximately 4-fold after acetaminophen challenge in the corn oil group. In contrast, those levels in beef tallow and olive oil groups increased 2-fold compared to control groups. When compared with acetaminophen-injected groups, animals in the corn oil group showed approximately 2-fold higher concentrations of marker enzymes than other groups.

### TBARS and GSH concentrations

Compared with control groups, lipid peroxidation measured by TBARS of acetaminophen-injected groups were significantly higher in animals fed corn oil (Figure 1C). In contrast to TBARS levels, hepatic GSH levels were significantly higher in animals fed olive oil and beef tallow compared to corn oil (Figure 1D).

### Protein expression of CYP 2E1

**Figure 2:**
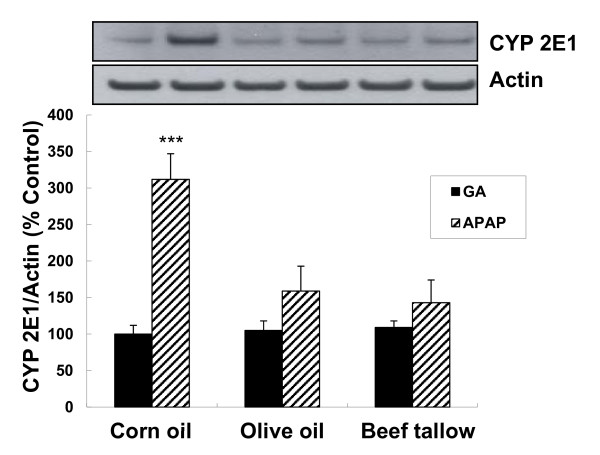
**Immunoblot analysis for cytochrome P-450 2E1 (CYP 2E1) in hepatic microsomes**. Microsomal proteins were analyzed by Western blot with polyclonal antibody against CYP 2E1. All data were normalized to the actin content of the same sample. Each bar represents the mean ± SE (n = 6). *** p < 0.001 compared with gum arbic vehicle and APAP-treated groups by Wilcoson rank sum test.

Acute acetaminophen treatment significantly induced the protein expression of CYP 2E1 in corn oil fed animals. However, slight increase in CYP 2E1 protein was observed in both of olive oil and beef tallow fed groups (Figure 2).

### Fatty acid composition of liver microsomal membranes

Since the type and amount of dietary fat are reflected in the total fatty acid profile in liver microsomal membrane and thus reflect susceptibility to oxidative stress, fatty acid compositions were determined and compared in Table 2. The major fatty acids were palmitic acid (16:0), stearic acid (18:0), oleic acid (18:1n-9), linoleic acid (18:2n-6), and arachidonic acid (20:4n-6). Despite the differences in dietary lipids, there were no significant differences in the proportion of SFA. However, significant differences were observed in the proportions of unsaturated fatty acids among the three diet groups. In comparison with the corn oil diet, lower levels of linoleic acid (18:2n-6) and higher levels of oleic acids (18:1n-9 and 18:1n-7) were detected in rats fed olive oil and beef tallow diets. The beef tallow group showed a lower level of MUFA compared to the olive oil group but the values were not significantly different. The ratios of linoleic to oleic acids were approximately 4- to 5-fold higher in the group fed the corn oil diet compared with the other diets. The major change was in the ratio between linoleic acid and oleic acid. The concentration of arachidonic acid (20:4n-6) was no significantly different among the groups. The DBI levels were observed in this order: corn oil (1.450) > olive oil (1.347) > beef tallow (1.286).

**Table 2:** Fatty acid composition of phospholipids in liver microsomal membrane^1^

Fatty Acid	Corn oil	Olive oil	Beef tallow
	
		μmole %	
C14:0	0.25 ± 0.10	0.42 ± 0.17	0.25 ± 0.15
C16:0	19.9 ± 0.86	21.4 ± 2.39	23.8 ± 2.35
C16:1n-7	0.25 ± 0.20^b^*	1.19 ± 1.36^a^	0.95 ± 0.24^a^
C18:0	25.6 ± 3.22	23.5 ± 2.14	23.9 ± 2.63
C18:1n-9	3.27 ± 2.30^b^**	9.46 ± 3.97^a^	6.91 ± 0.51^a^
C18:1n-7	1.04 ± 0.54	2.67 ± 0.63	1.93 ± 0.59
C18:2n-6	13.5 ± 1.08^a^**	7.12 ± 0.39^b^	7.20 ± 0.56^b^
C18:2/C18:1^2^	3.13 ± 0.62^a^***	0.63 ± 0.16^b^	0.82 ± 0.09^b^
C18:3n-3	0.47 ± 0.53	0.26 ± 0.28	0.03 ± 0.07
C20:4n-6	28.0 ± 1.40	26.6 ± 2.61	26.1 ± 1.87
DBI^3^	1.450	1.347	1.286

^1^Values are means ± S.D.

^2^The ratios of linoleic acid to oleic acids

^3^The double bond index (DBI) equals the sum obtained by multiplying the fraction of each fatty acid times the number of double bonds contained and adding the total together [10].

* p < 0.05, ** p < 0.005, *** p < 0.0001 compared with beef tallow and olive oil fed groups by Kruskal-Wallis test. Different superscripts for each fatty acid mean significantly different levels among groups.

## Discussion

The present study indicates that diets high in SFA (beef tallow) or MUFA (olive oil) decreased *in vivo *susceptibility to hepatotoxicity compared to diets high in linoleic acid (corn oil). Animals fed corn oil diet had significantly elevated plasma GPT and GOT levels as well as hepatic TBARS and CYP 2E1 after acetaminophen intoxication. Although the mechanism is unclear, these effects may be related to the observed changes in membrane phospholipid composition. The major difference was between linoleic and oleic acids. In comparison with the corn oil diet, lower levels of linoleic acid and higher levels of oleic acids were observed in rats fed olive oil and beef tallow diets. Therefore, the ratios of linoleic to oleic acids and DBI were significantly higher in the group fed the corn oil diet compared with the other diets. These observations are in agreement with other researchers [2-4], who reported that linoleic acid content of dietary fats was related to ethanol-induced liver injury and this injury was reversed with a diet rich in SFA but not unsaturated fats. These effects were highly correlated with the changes in phospholipid composition of liver microsomes.

Fatty acid compositions of dietary oils are reflected into fatty acid compositions of membrane phospholipid, which are the strong biochemical indicators of dietary fatty acids [11]. Fatty acid compositions of three different tissues and, in particular, the MUFA and PUFA content are highly dependent on the fat composition of the diet by *de novo *synthesis or by elongation from fatty acids provided by the dietary fats [5,12-14]. Our results showed that a high level of linoleic acid in the diet induced an increase of linoleic acid with a concomitant decrease of oleic acid in liver phospholipids. In liver microsomal membranes from rats fed corn oil, levels of linoleic acid were elevated 2-fold compared to those found in beef tallow and olive oil fed rats and oleic acid showed opposite trends. Therefore, the ratios of linoleic to oleic acid were approximately 4- to 5-fold higher in corn oil diet compared with the other diets. These differences in membrane composition may alter enzyme activity and affect drug metabolism and toxicity by altering membrane conformation and fluidity [3,15]. Moussa *et al*. [14] reported that lymphocyte phospholipid preferentially stimulated the synthesis of MUFA, especially oleic acid, by increasing Δ9-desaturase activity rather than SFA uptake in SFA-rich coconut-oil diets. Some evidences have supported that suppressive synthesis of oleic acid by n-6 PUFA rich diet and inhibitory activity of Δ9-desaturase by an excess of linolenyl-CoA, which is metabolized from linoleic acid [14]. Recently mounting evidences have shown that diets with high oleic acid and low linoleic acid may be protective effects against diseases [5,13,16-19].

Models of drug-induced liver toxicity involve at least four different components, including 1) increased levels of CYP; 2) provision of substrate that generate free radicals; 3) targets susceptible to free radical damage; and 4) deletion of cellular antioxidants.

A toxic metabolite produced by the CYP system can cause liver toxicity. Especially CYP 2E1 is the rate-limiting and major enzyme form in acetaminophen oxidation and thereby hepatotoxicity using CYP 2E1-null mice [20,21]. Therefore, elevated CYP 2E1 and microsomal MFO activity in animals fed PUFA compared to SFA might account, in part, for the liver injury [22,23]. In our study, treatment with acute acetaminophen significantly induced CYP 2E1 levels by 3-fold in corn oil-fed rats, but not in olive oil- and beef tallow-fed rats. Arachidonic (20:4n-6) and γ-linolenic (18:3n-6) acids, but not from SFA or MUFA, induced necrosis by activation of Erk1/2 signaling in CYP 2E1-overexpressing cells [24]. This isoenzyme induction by PUFA is involved in the activation of N-acetyl-*p*-benzoquinone imine (NAPQI), an acetaminophen metabolite, and thus enhance acetaminophen toxicity [6]. Yoo *et al*. [25] reported that 20% corn oil or linoleic acid equivalent increased in the concentration of CYP 2E1 by 2- to 3-fold. Particularly, linoleic acid as well as the highly unsaturated, long chain fatty acids found in the fish oils [eicosapentaenoic acid (EPA) and docosahexaenoic acid (DHA)] have been shown to be important in this respect [23]. Recently, Ito *et al*. [26] reported that Western-style diet (41% fat) protected acetaminophen-induced injury such as elevated mRNA level of CYP 2E1, alanine aminotransferase and hepatic centrilobular injury. In a similar manner of acetaminophen, ethanol and arachidonic acid stimulated lipid peroxidation and hepatotoxicity by expressing CYP 2E1, which was apoptotic in HepG2 cells [27]. This hepatotoxicity was prevented by inhibitors of CYP 2E1 and antioxidants [27].

Dietary fat may alter the activity of CYP by altering its intramembrane positioning, which may increase its reactivity with substrates [8]. The fatty acid composition of the cell membrane is highly susceptible to dietary manipulation, and changes in fatty acid occurred in red blood cell membranes after 2 weeks in pigs [7] after switching to experimental diets. Berlin *et al*. [7] also found that (n-6) fatty acids increased the membrane fluidity more than (n-3) fatty acids.

PUFA are the principal chemical species in cell membranes that are vulnerable to free radical-initiated peroxidative damage. Increasing PUFA intake therefore raises the cell membrane PUFA content and peroxidizability. Linoleic acid oxidizes easily and generates cytotoxic products, such as linoleic acid hydroperoxides or 4-hydroxy-2-(E)-nonenal [28]. Feeding corn oil significantly elevated linoleic acid in liver membranes and markedly increased lipid peroxidation products in rabbit livers when compared to beef tallow [29]. Using an *in vitro *hepatocyte culture model, Lamb *et al*. [30] showed that ethanol-dependent decreases in cell function were potentiated by linoleic acid and reduced by SFA (stearic acid). They proposed that PUFA but not SFA in membrane phospholipids were critical targets of reactive oxygen species (ROS) such as hydroxyl radicals and superoxide anions. In comparative toxicity studies of various fatty acids, all fatty acids at very high concentrations (200 - 600 μM) induced ROS production by activation of NADPH oxidase and apoptosis and necrosis in Jurkat and Raji cells, which were derived from human lymphocytes [31]. ROS production was accelerated in orders of linoleic acid > oleic acid > stearic acid. Oleic acid was less potent than linoleic acid in stimulating ROS production in both of human lymphocytes [31]. The protective mechanism of SFA and MUFA is due to reduced concentration of PUFA in the liver and diminished the availability of substrate for lipid peroxidation [2,3,18,19,22].

Oleic acid may act as a competitive inhibitor of PUFA oxidation. Although one double bond can be oxidized, its rate of oxidation is much less than the divinyl methane groups in PUFA. A divinyl methane group is a pair of unsaturated bonds separated by two saturated bonds. Oleic acid contains one of these groups, but PUFA contain from one to five. It has been suggested that the monohydroxy products generated during oxidation of oleic acid or other MUFA may be more stable and less capable of subsequent oxidation. Another possible mechanism is that oleic acid exerts antioxidant effects by increasing the delay prior to rapid oxidation of isolated LDL [32]. Fatty acids (100 μmol/L) supplementation with stearic acid [33], oleic acid, but not γ-linolenic acid (18:3n-6) [34] provided protection against H_2_O_2_-induced cytotoxicity, such as ROS and lipid hydroperoxide production [35] and lactate dehydrogenase (LDH) release [34], and from oxidized-LDL-induced endothelial dysfunction in **porcine aortic endothelial cells (PAEC)**. Diniz et al. [36] reported that there was no difference of LDH leakage in SFA- and PUFA-fed rats for 5 weeks, showing that PUFA diet resulted in the highest levels of myocardial lipoperoxide and lipid hydroperoxide and decreased superoxide dismutase and catalase activities. Using PAEC *in vitro*, treatment with 100 μmol/L oleic acid (beef tallow has more oleic acid than corn oil and soybean oil) attenuated H_2_O_2_-mediated LDH release but the same dose of linoleic acid and linolenic acid potentiated cytotoxicity [34]. In addition, olive oil contains phenolic antioxidants, such as tyrosol [2-(4-hydroxyphenyl)ethanol]. Some studies suggest that these compounds protect against LDL oxidation [37] and counteract the reactive oxygen metabolite-mediated cellular damage by improving *in vivo *antioxidant defenses [38].

It is unlikely that a change in SFA makes a difference. SFA compete very poorly for the sites occupied by linoleic acid and other PUFA. Thus, the reduced toxicity is likely to be due to increased MUFA content because this is a more favorable competitor. Precursors compete for esterification into membrane phospholipids in this order: HUFA > PUFA > MUFA > SFA. According to Lands model [39], PUFA are highly competitive, whereas MUFA and SFA are poor competitors. Saturation with n-6 PUFA and HUFA occurs in the range of 1-2% dietary energy. The amount of n-6 PUFA in the AIN 93G diet is about 9% of energy (60% linoleic acid × 15% fat of total energy). Therefore, the standard diet provides linoelic acid at more than 4-fold saturation. This probably explains why no change in arachidonic acid was observed in this experiment. To effectively change the ratio of oleic acid relative to linoleic acid, it may be necessary to decrease the intake of linoleic acid to 1-2% of energy while maintaining MUFA.

Animals fed corn oil tended to increase arachidonic acid compared to the other groups. This higher proportion of arachidonic acid in corn oil groups may be attributed to the high content of substates, *i.e*. linoleic acid, for desaturation and elongation in corn oil. Linoleic acid is rapidly incorporated into tissues and complex lipids and elongated and desaturated to arachidonic acid. Conversely, higher intake of oleic acid may displace linoleic acid from membranes and favor oxidation rather than conversion to arachidonic acid. After being released from liver microsomal membrane, PUFA activate phospholipase A2 and favor oxidation of arachidonic acid by cytochrome P450 system [40]. In our previous study [12], olive oil promoted an increase in EPA and DHA in phospholipids. These long chain fatty acids are derived from α-linolenic acid through Δ6-desaturation and elongation steps. The differences in the fatty acid composition may be attributed to increased hepatic turnover and the subsequent release of long chain PUFA. It is possible that animals fed corn oil have constitutively low Δ6-desaturase activities because desaturation, not elongation, is the rate-limiting step in the n-3 biosynthetic pathway [41]. Moreover, EPA and DHA have been reported to induce prostaglandin E3 (PGE3) and thromboxane A3 (TXA3), a weak platelet aggregator and vasoconstrictor and leukotriene B5 (LTB5), a weak inducer of inflammation and chemotaxis by inhibiting arachidonic acid conversion by cyclooxygenase with substrate regulation [42,43]. Concurrently, these n-3 fatty acids reduced production of thromboxane A2 (TXA2), a potent platelet aggregator and vasoconstrictor and leukotriene B4 (LTB4), a potent inducer of inflammation and leukocyte chemotaxis and adherence [43]. Nanji and his colleagues reported that fish oil [44] and olive oils [45] significantly reduced TXB2 and PGE2 levels in neutrophils and leukocytes, respectively. TXB2:PGE2 ratio was positively associated with hepatotoxicity [46,47]. In this way, n-3 PUFAs yield anti-inflammatory, anti-thrombotic, and vasodilatory properties, whereas high intake of linoleic acid accrues the opposite physiological state.

Reduction of arachidonic acid may decrease the formation of eicosanoids and promote less free radical generation. Nanji et al. [2] also suggested that this hepatoprotective effect might be caused by diminishing the availability of substrate for lipid peroxidation in the liver with substitution of PUFA with SFA. Therefore, decreased levels of this peroxidizable fatty acid may account, in part, for the absence of liver injury. Moreover, arachidonic acid might contribute to increased oxidation in animals fed corn oil rich in linoleic acid [29]. They also suggested that corn oil-fed animals had low scavenging capacity to quench free radicals and the low resistance to peroxidation when compared to animals fed more SFA diets. Nanji et al. [47,48] showed that both PGE_2 _and 6-keto-prostaglandin F1α were reduced in liver nonparenchymal cell, which was related to pathological liver injury in rats fed corn oil and ethanol. Acetaminophen-induced liver injury *in vivo *was significantly reduced by prostacyclin which is considered to be cytoptotective [26,47,48].

Some evidences [1,4,18,19] have suggested that PUFA can induce hepatotoxicity by increasing lipid peroxidation. In view of significantly higher values of biochemical markers of liver damage (*e.g*. increased plasma levels of GPT, GOT and TBARS) as well as decreased GSH levels, it is supported that PUFA can induce liver injury. Our results showed that hepatic GSH, which was measured after intoxication with acetaminophen, was significantly lower in corn oil fed animals than counterpart of those groups. GSH can be trapped with NAPQI, which was formed by acetaminophen oxidation in human liver [6]. Since GSH depletion was the critical marker in acetaminophen-induced hepatotoxicity, prevention of GSH depletion may be important in protection against acetaminophen-induced hepatotoxicity [49]. GSH depletion can markedly compromise the overall antioxidant system because glutathione metabolism appears to be a crucial component of the overall defense against oxidative stress within a cell.

## Conclusions

In conclusion, the present study confirms that drug-induced liver injury is promoted by substitution of PUFA from rich sources such as corn oil for fatty acids derived from beef tallow and olive oil. However, it is unlikely that the protection afforded by these dietary fats is effectively with PUFA for esterification into phospholipids. No change occurred in composition of SFA in our study. Instead, a major shift between linoleic acid and oleic acid occurred. Our data suggest that diets with high MUFA protect against the hepatotoxicity of acetaminophen by affecting the fatty acid composition of membrane phospholipids, thereby reducing susceptibility to free radical damage. A clear demonstration that the fatty acids present in beef tallow and olive oil help reduce this kind of injury could lead to better understanding of what constitutes a healthy diet. These findings help understand the effects of type of dietary fat on the nutritional management of drug-induced hepatotoxicity. Other types of dietary fats and/or chronic drug-induced hepatotoxicity need to be investigated for future study.

## Competing interests

The authors declare that they have no competing interests.

## Authors' contributions

JH and YC designed the experiment and wrote the manuscript. JH, JP, SK, HC, ES, and HH conducted data collection and analyses and reviewed the manuscript. All authors made critical comments during study design and preparation of manuscript and have given their final approval of the version to be published.
